# *Klebsiella pneumoniae* pneumonia in patients with rheumatic autoimmune diseases: clinical characteristics, antimicrobial resistance and factors associated with extended-spectrum β-lactamase production

**DOI:** 10.1186/s12879-021-06055-1

**Published:** 2021-04-17

**Authors:** Yang Liu, Yecheng Liu, Jiayuan Dai, Anlei Liu, Yi Li, Jun Xu, Xuezhong Yu, Jihai Liu, Huadong Zhu

**Affiliations:** Department of Emergency Medicine, Peking Union Medical College Hospital, Peking Union Medical College, Chinese Academy of Medical Sciences, 1 Shuaifuyuan, Dongcheng District, Beijing, China

**Keywords:** *Klebsiella pneumoniae* pneumonia, Rheumatic autoimmune diseases, ESBL, Mortality, Corticosteroids, Antimicrobial resistance

## Abstract

**Background:**

Over the past decades, *Klebsiella pneumoniae* (*K. pneumoniae*) infections have been increasing and affected immunocompromised patients nosocomially and communally, with extended-spectrum β-lactamase (ESBL) production becoming a major concern. Patients with rheumatic autoimmune diseases, mostly receiving immunosuppressive therapy, are vulnerable to various infections, including *K. pneumoniae*. However, few have investigated *K. pneumoniae* infections in this specific population. This study aimed to identify factors associated with ESBL production and mortality of *K. pneumoniae* pneumonia among patients with rheumatic autoimmune diseases in the Emergency Department.

**Methods:**

We retrospectively investigated patients with rheumatic diseases who were diagnosed with *K. pneumoniae* pneumonia. The diagnosis of *K. pneumoniae* pneumonia was based on clinical manifestations, radiological findings and microbiological testing results. Prognostic factors and risk factors for ESBL production were determined with univariate and multivariate logistic regression analysis. Empirical therapy and antimicrobial susceptibility data were also collected.

**Results:**

Of 477 *K. pneumoniae* pneumonia patients, 60 were enrolled into this study. The in-hospital mortality was 28.3%. Septic shock, ICU admission, the need for mechanical ventilation and change of antibiotics due to clinical deterioration, all related to mortality, were included as unfavorable clinical outcomes. Multivariate analysis suggested that ESBL production (OR, 6.793; *p* = 0.012), initial PCT ≥ 0.5 ng/ml (OR, 5.024; *p* = 0.033) and respiratory failure at admission (OR, 4.401; *p* = 0.046) predicted increased mortality. ESBL production was significantly associated with dose of corticosteroids (OR, 1.033; *p* = 0.008) and CMV viremia (OR, 4.836; *p* = 0.032) in patients with rheumatic autoimmune diseases. Abnormal leukocyte count (OR, 0.192; *p* = 0.036) was identified as a protective factor of ESBL-producing *K. pneumoniae* pneumonia. The most commonly used empirical antibiotic was ceftazidime, while most isolates showed less resistance to carbapenems and amikacin in susceptibility testing.

**Conclusions:**

*K. pneumoniae* pneumonia could be life-threatening in patients with rheumatic autoimmune diseases. Our findings suggested that ESBL production, initial PCT ≥ 0.5 ng/ml and respiratory failure at admission were independent factors associated with poor prognosis. Dose of corticosteroids and CMV viremia, predicting ESBL production in *K. pneumoniae* pneumonia, may help make individualized antibiotic decisions in clinical practice.

## Introduction

*Klebsiella pneumoniae* (*K. pneumoniae*) is classically an opportunistic bacterial pathogen. When first recognized, it was usually encountered in hospital-acquired infections, and has been extensively studied in hospitalized individuals [1–3]. In the past decades, community-associated infections with *K. pneumoniae* have been increasing, and pose a threat especially to immunocompromised patients. Individuals with rheumatic autoimmune diseases, mostly receiving immunosuppressive therapy, are vulnerable to this opportunistic infection. *K. pneumoniae* causes various infections including pneumonia. Mortality in *K. pneumoniae* pneumonia (*Kp* pneumonia) has been reported as high as 50% [4, 5]. It is suggested that outcomes in patients with Gram-negative bacterial (GNB) infections can be significantly improved if timely appropriate antibiotic therapy is provided [6]. While β-lactam antibiotics, especially third-generation cephalosporins, are frequently used to treat GNB infections including *Kp* pneumonia, a dramatic increase in antibiotic resistance has happened over the past decades, with resistance to β-lactams having the greatest impact on therapeutic effectiveness [4]. Thus, extended-spectrum β-lactamases (ESBL) producing *K. pneumoniae*, able to hydrolyze the antibiotic β-lactam ring, has become a major concern in clinical practice.

ESBL production in *K. pneumoniae* is generally believed to be associated with unfavorable outcomes, including extended length of hospital stay, increased mortality and in-hospital expenses, which has been investigated in *K. pneumoniae* bacteremia and healthcare-associated pneumonia [7–10]. However, some other studies did not demonstrate the correlation between ESBL production and mortality [11, 12]. With regard to ESBL acquisition, risk factors include previous use of antibiotics, prolonged hospitalization, ICU stay and mechanical ventilation, as has been reported previously [4, 13]. Nevertheless, limited data are available about antimicrobial resistance and clinical outcomes of *Kp* pneumonia among patients with rheumatic diseases.

In the present study, we retrospectively investigated clinical characteristics and outcomes of *Kp* pneumonia, as well as risk factors for ESBL-producing *K. pneumoniae*, among patients with rheumatic autoimmune diseases.

## Methods

### Study population

This study investigated patients with rheumatic autoimmune diseases who were admitted from Emergency Department and diagnosed with pneumonia caused by *K. pneumoniae* between January 2013 and December 2019 at Peking Union Medical College Hospital, a tertiary care hospital in Beijing, China.

### Microbiology

Microbiology testing was carried out in the Department of Laboratory Medicine at Peking Union Medical College Hospital. Antimicrobial susceptibility testing was carried out by broth microdilution methods per Clinical and Laboratory Standards Institution (CLSI) guidelines [14], and the antimicrobial agents tested included amoxicillin-clavulanic acid, amikacin, aztreonam, cefepime, cefperazone-sulbactam, ceftazidime, ceftriaxone, cefuroxime, ciprofloxacin, ertapenem, gentamicin, imipenem, levofloxacin, meropenem, minocycline, piperacillin-tazobactam, sulfamethoxazole trimethoprim and tigecycline. Susceptibility testing results were interpreted using latest CLSI clinical breakpoints [15]. Production of ESBLs was screened using the disk diffusion method as per CLSI document M100-S29 [15].

“Intermediate” and “resistant” strains in antimicrobial susceptibility testing were defined as “non-susceptible” isolates. Patients with more than one culture of respiratory tract specimens, at least one strain showing ESBL production, were included in the ESBL-positive group. Carbapenem-resistant *K. pneumoniae* isolates were defined as those resistant to any of the following carbapenems: ertapenem, meropenem or imipenem (MIC ≥4 μg/mL).

### Data collection and definitions

Medical records were retrospectively reviewed and the following data were extracted from the hospital’s electronic database: (1) clinical characteristics, including age, sex, underlying disease, immunosuppressive medication, comorbidity, clinical manifestation and disease course; (2) laboratory findings, including blood leukocyte count, urea, C-reactive protein, lactate dehydrogenase, procalcitonin (PCT) and virus PCR assay; (3) microbiological results, including smear and culture of respiratory tract specimen, ESBL production and antimicrobial susceptibility pattern.

Since isolation of *K. pneumoniae* from sputum may not be indicative of pneumonia, the diagnosis of *Kp* pneumonia was based on clinical manifestations (such as fever, “currant jelly” sputum or pleuritic chest pain), radiological findings (such as typical alveolar consolidation on chest computed tomography), and microbiological test results.

The daily dosage of corticosteroids was expressed as the prednisone equivalent (1 mg of prednisone equals 0.8 mg of methylprednisolone which equals 1 mg of prednisolone). Corticosteroid pulse therapy referred to intravenous methylprednisolone at dosages of 500-1000 mg for 3–5 consecutive days. Cytomegalovirus (CMV) viremia was defined as plasma CMV-DNA > 500 copies/ml by quantitative PCR. Respiratory failure at admission was defined as admission PaO_2_ lower than 60 mmHg with or without PaCO_2_ higher than 50 mmHg while breathing room air [16].

### Statistical analysis

Statistical analyses were performed using Stata 14.0 SE or GraphPad Prism 6.0. The variables in the datasets were presented as mean ± SD or number and proportion of the total (%). Continuous variables were analyzed by Mann-Whitney U test. Categorical variables were analyzed with Chi-square test or Fisher’s exact test, as appropriate. Given the number of cases available, variables were carefully chosen for multivariate analysis. With clinical relevance taken into account, variables found significant (*p* < 0.05) in univariate analysis were selected by the least absolute shrinkage and selection operator (LASSO) and were further examined in the multivariate logistic regression model.

### Ethical approval

This study conformed to the Declaration of Helsinki and was approved by the Medical Ethics Committee of Peking Union Medical College Hospital. Written informed consent for inclusion from each patient was waived by the Medical Ethics Committee of Peking Union Medical College Hospital because this was a retrospective study, and no study-related interventions were included.

## Results

### Patient characteristics

During the study period, 477 patients present to the Emergency Department were diagnosed with *Kp* pneumonia. Among these patients, 63 were identified as suffering from rheumatic autoimmune diseases. Three of them, treated as out-patients, were excluded from our analysis due to incomplete medical records. Within the included cohort (60 patients), 20 (33.3%) had systemic lupus erythematosus (SLE), 9 (15%) had polymyositis or dermatomyositis (PM/DM), and 8 (13.3%) had vasculitis. Other underlying autoimmune disorders are listed in Table 1. Females accounted for 60% of all the cases, and 80% of patients had a history of hospitalization within the previous 90 days. Fifty-eight patients (96.7%) were receiving corticosteroids, among which 8 (13.3%) had been given pulse therapy within 1 month before *Kp* pneumonia. The mean duration of corticosteroid administration was 22.9 ± 46.2 months, and an average dose of 52.2 ± 42.6 mg/d equivalent prednisone was administered at the time of diagnosis. In combination with corticosteroids, immunosuppressants was used in 25 patients (41.7%). The ICU admission rate was 63.3%, and the in-hospital mortality rate was 28.3% in the study cohort (Table 2).

**Table 1:** Underlying rheumatic autoimmune diseases in patients with *Kp* pneumonia

Rheumatic autoimmune diseases	*N* = 60
SLE	20 (33%)
PM/DM	9 (15%)
Vasculitis	8 (13%)
Undifferentiated CTD	6 (10%)
pSS	5 (8%)
RA	4 (7%)
ASD	3 (5%)
SSc	2 (3%)
Primary APS	1 (2%)
Overlap syndrome	1 (2%)
PMR	1 (2%)

The case of overlap syndrome included features of SLE and PM

Data were presented as numbers (%)

*SLE* systemic lupus erythematosus, *PM/DM* polymyositis/dermatomyositis, *CTD* connective tissue disease, *pSS* primary Sjögren syndrome, *RA* rheumatoid arthritis, *ASD* adult Still’s disease, *SSc* systemic sclerosis, *APS* antiphospholipid syndrome, *PMR* polymyalgia rheumatica

**Table 2:** Clinical characteristics and comparisons between survivors and non-survivors

Variables	Total (*N* = 60)	Non-survivors (*N* = 17)	Survivors (*N* = 43)	*p* value
Age (years)	51.2 ± 17.9	47.2 ± 21.2	52.8 ± 16.4	0.465
Sex (female)	36 (60.0%)	11 (64.7%)	25 (58.1%)	0.640
Corticosteroid use	58 (96.7%)	16 (94.1%)	42 (97.7%)	0.490
Pulse therapy	8 (13.3%)	3 (17.6%)	5 (11.6%)	0.676
Duration of corticotherapy (months)	22.9 ± 46.2	36.8 ± 70.6	17.4 ± 31.5	0.913
Dose of corticosteroids (mg)	52.2 ± 42.6	51.2 ± 36.4	52.5 ± 45.2	0.944
Immunosuppressants	25 (41.7%)	9 (52.9%)	16 (37.2%)	0.265
Hospitalization within last 90 days	48 (80.0%)	13 (76.5%)	35 (81.4%)	0.726
Previous antimicrobial therapy within 30 days	52 (86.7%)	16 (94.1%)	36 (83.7%)	0.420
Ever-smokers	15 (25.0%)	2 (11.8%)	13 (30.2%)	0.192
Pulmonary diseases^a^	29 (48.3%)	8 (47.1%)	21 (48.8%)	1.000
Hypertension	22 (36.7%)	8 (47.1%)	14 (32.6%)	0.376
Diabetes mellitus	14 (23.3%)	4 (23.5%)	10 (23.3%)	1.000
Fever	45 (75.0%)	14 (82.4%)	31 (72.1%)	0.520
Cough/expectoration	49 (81.7%)	11 (64.7%)	38 (88.4%)	0.059
Initial body temperature (°C)	37.40 ± 0.99	37.44 ± 1.15	37.38 ± 0.94	0.722
Mean arterial pressure (mmHg)	90.9 ± 17.3	89.3 ± 18.1	91.6 ± 17.2	0.785
Respiratory failure at admission	26 (43.3%)	11 (64.7%)	15 (34.9%)	0.036
Blood leukocyte count (10^9^/L)	10.01 ± 5.60	9.89 ± 5.74	10.06 ± 5.61	0.994
Blood neutrophil count (10^9^/L)	8.69 ± 5.29	8.61 ± 5.35	8.72 ± 5.34	0.993
Blood lymphocyte count (/μL)	867 ± 978	877 ± 1300	863 ± 836	0.450
Albumin (g/L)	29.8 ± 4.8	28.7 ± 5.1	30.2 ± 4.6	0.352
Alanine aminotransferase (U/L)	82.3 ± 282.8	48.8 ± 53.0	95.8 ± 333.8	0.847
Creatinine (μmol/L)	83.8 ± 81.3	81.9 ± 59.3	84.5 ± 89.3	0.854
Urea (mmol/L)	9.41 ± 6.65	12.33 ± 7.72	8.22 ± 5.86	0.011
C-reactive protein (mg/L)	71.2 ± 63.6	78.3 ± 67.8	67.1 ± 62.0	0.407
Lactate dehydrogenase (U/L)	537.6 ± 424.8	710.0 ± 588.5	425.5 ± 227.2	0.146
PCT (ng/ml)				0.012
< 0.5	39 (65.0%)	7 (41.2%)	32 (74.4%)	
0.5–2	9 (15.0%)	2 (11.8%)	7 (16.3%)	
2–10	9 (15.0%)	6 (35.3%)	3 (7.0%)	
> 10	3 (5.0%)	2 (11.8%)	1 (2.3%)	
CMV viremia	19 (31.7%)	8 (47.1%)	11 (25.6%)	0.107
ESBL production	22/51 (43.1%)	10/14 (71.4%)	12/37 (32.4%)	0.012
Carbapenem resistance	5 (8.3%)	2 (11.8%)	3 (7.0%)	0.616
Bacteremia	11 (18.3%)	5 (29.4%)	6 (14.0%)	0.265
*Outcomes*				
Change of antibiotics due to clinical deterioration	22 (36.7%)	12 (70.6%)	10 (23.3%)	0.001
Septic shock	14 (23.3%)	10 (58.8%)	4 (9.3%)	< 0.001
ICU admission	38 (63.3%)	16 (94.1%)	22 (51.2%)	0.002
Mechanical ventilation	26 (43.3%)	15 (88.2%)	11 (25.6%)	< 0.001
Length of ICU stay (days)	17.6 ± 19.5	16.2 ± 14.8	18.6 ± 22.7	0.939
Length of hospital stay (days)	27.5 ± 20.9	20.5 ± 17.5	30.4 ± 21.7	0.103
In-hospital mortality	17 (28.3%)			

Data were presented as mean ± SD or numbers (%)

^a^Pulmonary diseases included interstitial lung disease (20 cases), chronic obstructive pulmonary disease (1 case), asthma (1 case), bronchiectasis (1 case), inactive pulmonary tuberculosis (2 cases), diffuse alveolar hemorrhage (2 cases) and pulmonary arterial hypertension (2 cases)

### Prognostic factors

Unfavorable clinical outcomes included in-hospital death, as well as septic shock, ICU admission, the need for mechanical ventilation and change of antibiotics due to clinical deterioration, which were strongly related to mortality. Thus, in order to identify prognostic factors for *Kp* pneumonia among patients with rheumatic autoimmune diseases, 43 survivors were compared with 17 non-survivors. As is shown in Table 2, respiratory failure at admission was significantly more prevalent in non-survivors (*p* = 0.036). The level of blood urea (*p* = 0.011) and PCT (*p* = 0.012) were also associated with prognosis. ESBL production (*p* = 0.012) was related to increased mortality. Multivariate logistic regression suggested ESBL production (OR, 6.793; 95% CI, 1.533–30.102), initial PCT ≥ 0.5 ng/ml (OR, 5.024; 95% CI, 1.138–22.189) and respiratory failure at admission (OR, 4.401; 95% CI, 1.030–18.808) were independent predictors of unfavorable outcomes (Table 3).

**Table 3:** Factors associated with in-hospital mortality from *Kp* pneumonia

Variables	Univariate			Multivariate		
	Odds ratio	95% CI	*p* value	Odds ratio	95% CI	*p* value
ESBL production	4.800	1.341–17.186	0.016	6.793	1.533–30.102	0.012
Initial PCT ≥ 0.5 (ng/ml)	4.156	1.272–13.581	0.018	5.024	1.138–22.189	0.033
Respiratory failure at admission	3.422	1.056–11.092	0.040	4.401	1.030–18.808	0.046

*CI* confidence interval

### Empirical therapy and antimicrobial susceptibility pattern of *K. pneumoniae* isolates

**Fig. 1:**
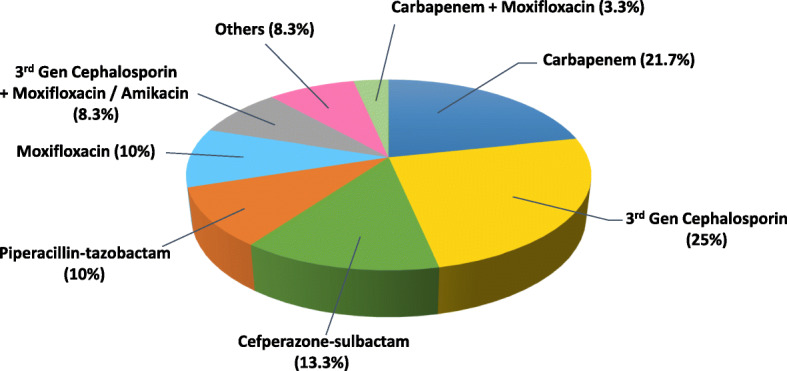
Empirical antimicrobial therapy administered to patients with *Kp* pneumonia

The most common empirical antibiotic was ceftazidime (*n* = 18, 30%). Thirteen patients were administered with ceftazidime alone (21.7%) and 5 were treated in combination with amikacin or moxifloxacin (8.3%). Imipenem (*n* = 9, 15%) and cefperazone-sulbactam (*n* = 8, 13.3%), following ceftazidime, were also frequently used as empirical therapy. Figure 1 demonstrated antimicrobial treatments that were administered empirically.

**Fig. 2:**
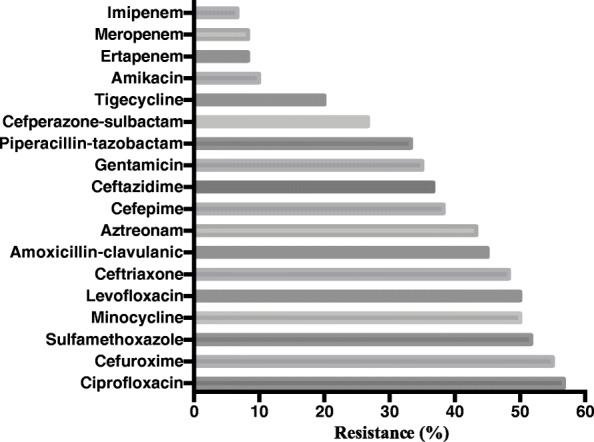
Antimicrobial resistance pattern of *K. pneumoniae* isolates

According to antimicrobial susceptibility testing, most isolates were less resistant to carbapenems (6.7–8.3%) and amikacin (10.0%), while highly resistant to ciprofloxacin (56.7%). Ceftazidime resistance was observed in 36.7% isolates of *K. pneumoniae* (Table 4, Fig. 2).

**Table 4:** Antimicrobial susceptibility pattern of *K. pneumoniae* isolates

Antibiotics	Susceptibility (*N* = 60)	Resistance
Imipenem	56 (93.3%)	6.7%
Meropenem	55 (91.7%)	8.3%
Ertapenem	55 (91.7%)	8.3%
Amikacin	54 (90.0%)	10.0%
Tigecycline	48 (80.0%)	20.0%
Cefperazone-sulbactam	44 (73.3%)	26.7%
Piperacillin-tazobactam	40 (66.7%)	33.3%
Gentamicin	39 (65.0%)	35.0%
Ceftazidime	38 (63.3%)	36.7%
Cefepime	37 (61.7%)	38.3%
Aztreonam	34 (56.7%)	43.3%
Amoxicillin-clavulanic acid	33 (55.0%)	45.0%
Ceftriaxone	31 (51.7%)	48.3%
Levofloxacin	30 (50.0%)	50.0%
Minocycline	30 (50.0%)	50.0%
Sulfamethoxazole trimethoprim	29 (48.3%)	51.7%
Cefuroxime	27 (45.0%)	55.0%
Ciprofloxacin	26 (43.3%)	56.7%

Strains showing “intermediate” in antimicrobial susceptibility testing were included as “non-susceptible” isolates

Resistance (%) = 1- susceptibility (%)

### Factors associated with ESBL-positive *Kp* pneumonia

As ESBL production was associated with mortality, we further investigated risk factors for ESBL-producing *Kp* pneumonia. In this study, 23 patients had ESBL-positive *Kp* pneumonia, while 32 had ESBL-negative *Kp* pneumonia (Table 5). Cases with *K. pneumoniae* strains resistant to carbapenems (*n* = 5) were excluded from the analysis. At the time of diagnosis, patients in ESBL-positive group were receiving higher doses of corticosteroids, as compared to those in ESBL-negative group (*p* = 0.015). Blood leukocyte count was also related to ESBL production (*p* = 0.035). The number of lymphocytes were significantly lower in patients with ESBL-producing *Kp* pneumonia (*p* = 0.015). However, no significant difference was observed in the number of neutrophils between the two groups. Patients with CMV viremia (*p* = 0.009) were more likely to be infected with ESBL-producing *K. pneumoniae*. Whether antibiotics were received within previous 30 days did not show a significant difference in ESBL production in this cohort. By multivariate analysis, dose of corticosteroids (OR, 1.033; 95% CI, 1.008–1.059) and CMV viremia (OR, 4.836; 95% CI, 1.142–20.480) were identified as independent variables associated with ESBL production, while abnormal leukocyte count (OR, 0.192; 95% CI, 0.041–0.901) was an independent protective factor of ESBL-positive *Kp* pneumonia (Table 6).

**Table 5:** Characteristics of patients with rheumatic diseases and pneumonia caused by *K. pneumoniae* with or without ESBL production

Variables	ESBL-positive (*N* = 23)	ESBL-negative (*N* = 32)	*p* value
Age (years)	48.0 ± 14.6	54.2 ± 18.0	0.096
Sex (female)	14 (60.9%)	17 (53.1%)	0.568
Duration of corticotherapy (months)	17.5 ± 37.0	29.3 ± 54.2	0.444
Dose of corticosteroids (mg)	72.3 ± 53.3	37.6 ± 28.4	0.015
Hospitalization within last 90 days	20 (87.0%)	23 (71.9%)	0.182
Previous antimicrobial therapy within 30 days	22 (95.7%)	25 (78.1%)	0.120
Diabetes mellitus	5 (21.7%)	9 (28.1%)	0.592
Fever	19 (82.6%)	23 (71.9%)	0.355
Cough/expectoration	18 (78.3%)	27 (84.4%)	0.726
Respiratory failure at admission	10 (43.5%)	14 (43.8%)	0.984
Blood leukocyte count (10^9^/L)			0.035
< 4	3 (13.0%)	4 (12.5%)	
4–10	14 (60.9%)	9 (28.1%)	
> 10	6 (26.1%)	19 (59.4%)	
Blood neutrophil count (10^9^/L)	8.17 ± 5.99	9.13 ± 5.03	0.246
Blood lymphocyte count (/μL)	516 ± 291	1143 ± 1255	0.015
Albumin (g/L)	28.9 ± 4.8	30.2 ± 4.9	0.259
Urea (mmol/L)	9.71 ± 5.61	8.99 ± 7.37	0.187
C-reactive protein (mg/L)	66.0 ± 59.8	79.0 ± 69.7	0.703
PCT (ng/ml)			0.899
< 0.5	15 (65.2%)	20 (62.5%)	
0.5–2	3 (13.0%)	6 (18.8%)	
2–10	4 (17.4%)	4 (12.5%)	
> 10	1 (4.4%)	2 (6.2%)	
CMV viremia	12 (52.2%)	6 (18.8%)	0.009

Data were presented as mean ± SD or numbers (%)

**Table 6:** Risk factors associated with ESBL-positive *Kp* pneumonia in patients with rheumatic autoimmune diseases

Variables	Univariate			Multivariate		
	Odds ratio	95% CI	*p* value	Odds ratio	95% CI	*p* value
Dose of corticosteroids (mg)	1.024	1.005–1.042	0.011	1.033	1.008–1.059	0.008
Abnormal leukocyte count^a^	0.252	0.081–0.785	0.017	0.192	0.041–0.901	0.036
Blood lymphocyte count (/μL)	0.198	0.038–1.036	0.055			
CMV viremia	4.727	1.414–15.809	0.012	4.836	1.142–20.480	0.032

*CI* confidence interval

^a^Abnormal leukocyte count was defined as leukocyte count < 4 × 10^9^/L or leukocyte count > 10 × 10^9^/L.

## Discussion

*K. pneumoniae*, as a clinically relevant pathogen, has been well studied in hospitalized patients [4]. Individuals with rheumatic autoimmune diseases, mostly receiving corticosteroids, are rendered immunocompromised and may also be susceptible to *K. pneumoniae*. However, few have investigated *Kp* pneumonia in this specific population. In this study, we comprehensively reviewed the clinical and laboratory findings of *Kp* pneumonia in patients with rheumatic autoimmune diseases.

Corticosteroid use comes with a number of well-established risks including infections. It has been reported a daily dose of 5 mg equivalent prednisone increases the risk for hospitalized pneumonia, with a higher risk at doses greater than 10 mg daily [17]. A meta-analysis of 42 observational studies in patients with rheumatoid arthritis (RA) or inflammatory polyarthritis found that the use of systemic corticosteroid therapy was associated with an increased risk of infection in a dose-dependent manner [18]. The duration of corticosteroid therapy is also important, but less well-defined [19]. In our cohort, a daily dose of 52.2 ± 42.6 mg equivalent prednisone, which was a relatively high dose, was administered prior to the diagnosis of *Kp* pneumonia. The duration of corticosteroid administration was 22.9 ± 46.2 months. Nevertheless, neither the dose nor the duration of corticosteroid therapy was associated with prognosis of *Kp* pneumonia in patients with rheumatic autoimmune diseases.

In the attempt to identify prognostic factors, our data showed that initial PCT ≥ 0.5 ng/ml significantly predicted in-hospital death. PCT is a useful biomarker to distinguish bacterial infection from other causes of infection. In a meta-analysis of 14 trials and 4211 patients with respiratory tract infection, initial PCT levels were associated with an increased risk of treatment failure and mortality, especially in the emergency department setting [20]. An observational study also found that PCT was related to illness severity in patients with severe pneumonia, including community-acquired pneumonia (CAP), ventilator-associated pneumonia (VAP) and hospital-acquired pneumonia (HAP) [21]. Therefore, our findings among patients with rheumatic autoimmune diseases are consistent with previous studies in general population. In the present study, respiratory failure at admission (PaO_2_ < 60 mmHg) was developed in 43.3% of the patients and was found to be an independent prognostic factor of *Kp* pneumonia in patients with rheumatic autoimmune diseases. Pneumonia Severity Index (PSI), which includes PaO_2_ < 60 mmHg as a parameter, is an effective tool in assessing severity of illness in patients with CAP [22]. Another observational study showed that low PaO_2_ in the first 24 h after admission was associated with in-hospital mortality in ICU patients [23]. The rationale could be that an abnormally low level of oxygen in blood (hypoxemia) may fail to meet the metabolic demands of certain tissues, which would adversely affect multiple organs and eventually lead to poor prognosis. ESBL production in *K. pneumoniae* is one of the important mechanisms for its antibiotic resistance. Infections caused by ESBL-producing *K. pneumoniae* are associated with higher rates of treatment failure and increased mortality [7, 8, 10], which presents a threat especially to immunocompromised patients. In contrast, a recent study in neutropenic patients with bloodstream infection failed to show the contribution of ESBL production to mortality risk [12]. In our cohort with pulmonary infection, patients infected with ESBL-producing *K. pneumoniae* had a higher mortality rate compared to those with non-ESBL-producing *K. pneumoniae* infection. It is possible that ESBL production may potentially lead to delayed appropriate therapy or even treatment failure, which has a negative impact on patient clinical outcomes [6].

Factors associated with ESBL production were assessed among patients with rheumatic autoimmune diseases. Hospitalization and previous use of antibiotics are considered to be risk factors for drug-resistant infection mediated by ESBLs [12, 24]. However, in this study, with regard to hospitalization (within previous 90 days) and receipt of antibiotics (within previous 30 days), we have not found statistically significant differences in ESBL production. Instead, dose of corticosteroid therapy was an independent risk factor for ESBL-positive *Kp* pneumonia. It has been reported that corticosteroid use is correlated with ESBL production in nosocomial infections and bloodstream infections by several studies [25–27]. In this pneumonia cohort, the majority of the patients were receiving corticosteroids, and those administered with higher doses were more likely to be infected with ESBL-positive *K. pneumoniae*, which may be explained by hyperglycemia and impaired cellular immunity associated with corticosteroid use [28, 29]. High concentrations of corticosteroids can suppress signals mediated by pattern recognition receptors and cytokine receptors, and inhibit the production of B cells and T cells. In addition, interestingly, CMV viremia was also identified as a significant predictor of ESBL-positive *Kp* pneumonia. CMV infection and reactivation occurs frequently in immunocompromised patients and critically ill immunocompetent patients and has been reportedly associated with adverse clinical outcomes [30, 31]. The possible explanation for our findings may be that CMV viremia reflects more severe immunosuppression and critically ill condition, under which patients are more vulnerable to drug-resistant infections.

Empirical antimicrobial therapy included third-generation cephalosporins (primarily ceftazidime), carbapenems, followed by cefperazone-sulbactam and piperacillin-tazobactam. Ceftazidime is frequently used to treat *Kp* pneumonia in general population and is mostly effective in ESBL-negative infections. However, in patients with underlying autoimmune disorder, ESBL-positive infections could be prevalent, and empirical therapy with ceftazidime could result in treatment failure. According to susceptibility testing, most isolates were less resistant to carbapenems, which could make them the preferred options for *Kp* pneumonia. In clinical practice, carbapenems are frequently used in treating ESBL-associated infections and are recommended to be administered to immunocompromised patients (corticosteroid use > 15 mg of prednisone daily for > 2 weeks) [32]. However, overuse of carbapenems and subsequent selective pressure can contribute to the spread of carbapenem-resistant *K. pneumoniae*. Therefore, empirical antibiotics should be selected cautiously. In patients with rheumatic autoimmune diseases, our findings suggested dose of corticosteroids and CMV viremia, predicting ESBL production in *Kp* pneumonia, may help make individualized antibiotic decisions.

There are some potential limitations of this study. First, the sample size was relatively small. We included pneumonia patients with rheumatic autoimmune diseases who were admitted from Emergency Department. Since a few patients were transferred from other hospitals, they may have hospital-acquired pneumonia. Due to limited cases, we did not perform subgroup analysis based on where the pneumonia was acquired. Nevertheless, the data did provide a general picture in the Emergency setting. Second, it was a retrospective study performed in a single institution. Some medical records had missing data, which may affect the results. The susceptibility pattern of *K. pneumoniae* isolates may vary in other centers and regions. However, as the data were collected from one center, the testing method for susceptibility and ESBL detection was consistent. Third, when evaluating clinical outcomes, we mainly assessed in-hospital mortality but not long-term survival of *Kp* pneumonia patients with rheumatic autoimmune diseases.

## Conclusions

In summary, *Kp* pneumonia could affect immunocompromised individuals and is potentially fatal. In patients with rheumatic autoimmune diseases, initial PCT ≥ 0.5 ng/ml, respiratory failure at admission and ESBL production predicted increased mortality. The most important factors found to be associated with ESBL-producing *Kp* pneumonia were dose of corticosteroids and CMV viremia, while abnormal leukocyte count was identified as a protective factor of ESBL-positive *Kp* pneumonia in this specific population. Well-designed prospective studies are required to validate the current findings.

## Data Availability

The datasets analyzed during the current study are available from the corresponding author H.Z. or J.L. on reasonable request.

## Abbreviations

*Kp*: *Klebsiella pneumoniae*
ESBL: Extended-spectrum β-lactamase
GNB: Gram-negative bacteria
CLSI: Clinical and Laboratory Standards Institution
PCT: Procalcitonin
CMV: Cytomegalovirus
LASSO: Least absolute shrinkage and selection operator
SLE: Systemic lupus erythematosus
PM: Polymyositis
DM: Dermatomyositis
CTD: Connective tissue disease
pSS: Primary Sjögren syndrome
RA: Rheumatoid arthritis
ASD: Adult Still’s disease
SSc: Systemic sclerosis
APS: Antiphospholipid syndrome
PMR: Polymyalgia rheumatica
OR: Odds ratio
CI: Confidence interval
CAP: Community-acquired pneumonia
VAP: Ventilator-associated pneumonia
HAP: Hospital-acquired pneumonia
PSI: Pneumonia Severity Index
